# Using Wearable Technology to Quantify Physical Activity Recovery: Secondary Report From the AFTER (App-Facilitated Tele-Rehabilitation) Program for COVID-19 Survivors Randomized Study

**DOI:** 10.2196/43436

**Published:** 2023-03-20

**Authors:** Laura Churchill, Mary Morrow, Jacob J Capin, Sarah E Jolley, Kristine Hare, Samantha MaWhinney, Jennifer E Stevens-Lapsley, Kristine M Erlandson

**Affiliations:** 1 Department of Physical Medicine and Rehabilitation University of Colorado Anschutz Medical Campus Aurora, CO United States; 2 Department of Biostatistics and Informatics University of Colorado Anschutz Medical Campus Aurora, CO United States; 3 Department of Physical Therapy Marquette University Milwaukee, WI United States; 4 Eastern Colorado Veterans Affairs Geriatric Research Education and Clinical Center Aurora, CO United States; 5 Department of Medicine University of Colorado Anschutz Medical Campus Aurora, CO United States

**Keywords:** Fitbit, steps, COVID-19, hospitalization, rehabilitation, digital health intervention, physical activity, step count, mHealth application, tele-rehabilitation

## Abstract

**Background:**

Knowledge on physical activity recovery after COVID-19 survival is limited. The AFTER (App-Facilitated Tele-Rehabilitation) program for COVID-19 survivors randomized participants, following hospital discharge, to either education and unstructured physical activity or a telerehabilitation program. Step count data were collected as a secondary outcome, and we found no significant differences in total step count trajectories between groups at 6 weeks. Further step count data were not analyzed.

**Objective:**

The purpose of this analysis was to examine step count trajectories and correlates among all participants (combined into a single group) across the 12-week study period.

**Methods:**

Linear mixed models with random effects were used to model daily steps over the number of study days. Models with 0, 1, and 2 inflection points were considered, and the final model was selected based on the highest log-likelihood value.

**Results:**

Participants included 44 adults (41 with available Fitbit [Fitbit LLC] data). Initially, step counts increased by an average of 930 (95% CI 547-1312; *P*<.001) steps per week, culminating in an average daily step count of 7658 (95% CI 6257-9059; *P*<.001) at the end of week 3. During the remaining 9 weeks of the study, weekly step counts increased by an average of 67 (95% CI −30 to 163; *P*<.001) steps per week, resulting in a final estimate of 8258 (95% CI 6933-9584; *P*<.001) steps.

**Conclusions:**

Participants showed a marked improvement in daily step counts during the first 3 weeks of the study, followed by more gradual improvement in the remaining 9 weeks. Physical activity data and step count recovery trajectories may be considered surrogates for physiological recovery, although further research is needed to examine this relationship.

**Trial Registration:**

ClinicalTrials.gov NCT04663945; https://tinyurl.com/2p969ced

## Introduction

Low physical functioning and physical inactivity are observed among people who were hospitalized with severe COVID-19 [1,2]. Understanding how COVID-19–related hospitalization and subsequent rehabilitation impacts postdischarge physical activity trajectories may inform prognosis and care.

Few studies have detailed data on physical activity levels after SARS-CoV-2 infection and hospitalization. A large-scale population-based study found that those who previously reported performing aerobic and muscle strengthening activities that met or surpassed physical activity recommendations in the 2 years prior to the COVID-19 pandemic had a lower risk of infection, severe illness when infected, and COVID-19–related death [3]. Another longitudinal study found a link between persistent symptoms and significantly decreased self-reported walking times at 3 and 6 months after symptom onset when these walking times were compared to preillness estimates [4].

Wearable fitness trackers, including smartwatches, enable objective, continuous, long-term monitoring and have various applications in health care settings, such as health monitoring and medication adherence monitoring [5]. However, research examining the use of wearables to monitor COVID-19 survivors’ physical activity over the course of recovery is limited. To our knowledge, a study conducted by Hunter et al [1] is the only study that has used smartwatches to assess step count changes in COVID-19 survivors following critical care hospital admission. They found that, on average, participants took 4359 steps per day in the first month after discharge and that their average step counts increased by 37% between discharge and 3 months after hospitalization and by 82% at 1 year after discharge when compared to baseline [1].

The AFTER (App-Facilitated Tele-Rehabilitation) program for COVID-19 survivors was a pilot study that randomized participants to either education and unstructured physical activity or a telerehabilitation program following discharge for COVID-19–related hospital admissions [6]. Step count data were collected as a secondary outcome, which was specified a priori as the change in average daily step counts from baseline to 6 weeks. As reported in the primary trial paper, we found no significant differences in total step count trajectories between groups at 6 weeks [6]. Since little is known regarding step count recovery after COVID-19 illness, we sought to further investigate these data across the entire study period (12 weeks). The purpose of this analysis was to examine step count trajectories and correlates among all participants (combined into a single group) across the entire study period. We hypothesized that upon discharge, participants’ baseline step counts would be below those of healthy adults, with continued improvements observed across the 12-week study.

## Methods

### Data, Sample, and Outcome Measures

A detailed description of the AFTER study methods can be found elsewhere [6]. Briefly, the AFTER trial randomized participants (N=44) in a 2:1 ratio to either (1) one-on-one sessions (n=12) of telerehabilitation that were delivered remotely by a physical therapist (n=29) or (2) a comprehensive educational handout that covered domains of COVID-19 recovery, including physical activity, sleep, and cognitive health (n=15). Participants in the AFTER trial had SARS-CoV-2 confirmed via polymerase chain reaction testing, were aged ≥35 years, had a hospitalization that lasted for ≥24 hours, were within 6 weeks of hospital discharge, and had internet access. The exclusion criteria were unstable medical comorbidities that precluded exercise, current pregnancy, or concurrent physical therapy during the study period. All participants received the Fitbit Inspire 2 activity monitor (Fitbit LLC) and a Kindle Fire tablet (Amazon Inc) with preloaded Fitbit software and the Health in Motion app (Blue Marble Health), which provided physical function testing, a health diary, education, and exercises. Participants were instructed to wear the Fitbit on their wrist at all times (except when charging). Research team members checked the synced accounts and reminded participants to wear their Fitbit during the physical therapy sessions (experimental group) and weekly check-in calls (control group). Participants were provided with the passwords for their web-based accounts for activity monitoring; the research team also had access to these accounts. Step counts were calculated by using the standard Fitbit algorithm. Data from each participant's Fitbit account were downloaded and used in the data analysis.

The education and exercise components of the app were only prescribed to the intervention group, although participants in the control group could access educational modules and physical function testing on their own.

### Ethics Approval

The AFTER trial was approved by the University of Colorado Institutional Review Board (reference number: COMIRB 20-2415) and registered on ClinicalTrials.gov (trial number: NCT04663945).

### Statistical Analysis

We analyzed all participants, who were combined into a single group. Linear mixed models with random effects were used to model daily steps over the number of study days. Models with 0, 1, and 2 inflection points were considered, and the final model was selected based on the highest log-likelihood value. The final model included a random intercept and random effects on both the pre– and post–inflection point trajectories. All participants with any synced Fitbit activity data were included. Model robustness was evaluated by reviewing results after excluding records with a daily step count beyond 2 SDs from the participants’ overall average. The adjustment variables considered in the analysis included treatment group, sex, age, and BMI. Baseline demographics and COVID-19 hospitalization information were presented by treatment group and were compared by using a chi-square test or the Mood median test, as appropriate. All statistical analyses were performed in SAS 9.4 (SAS Institute Inc); we assumed a significance level of *P*<.05, and there was no adjustment for multiple comparisons. R software (R Foundation for Statistical Computing) was used for all data cleaning, summary statistics, and graphics.

## Results

Participants included 44 adults who enrolled between December 2, 2020, and July 2, 2021. Fitbit data were available for 41 participants, with a median of 78 (IQR 64-83) contributing days. Table 1 presents participant characteristics.

The median age of participants was 53 (IQR 44-61) years, and 46% (19/41) were female. The median number of days that participants spent in the hospital was 4 (IQR 2-8), and 22% (9/41) of participants were admitted to the intensive care unit. The average length of time between hospital discharge and enrollment was 3.5 weeks. The median number of steps for the cohort at baseline was 4928 (IQR 3083-7574).

**Figure 1:**
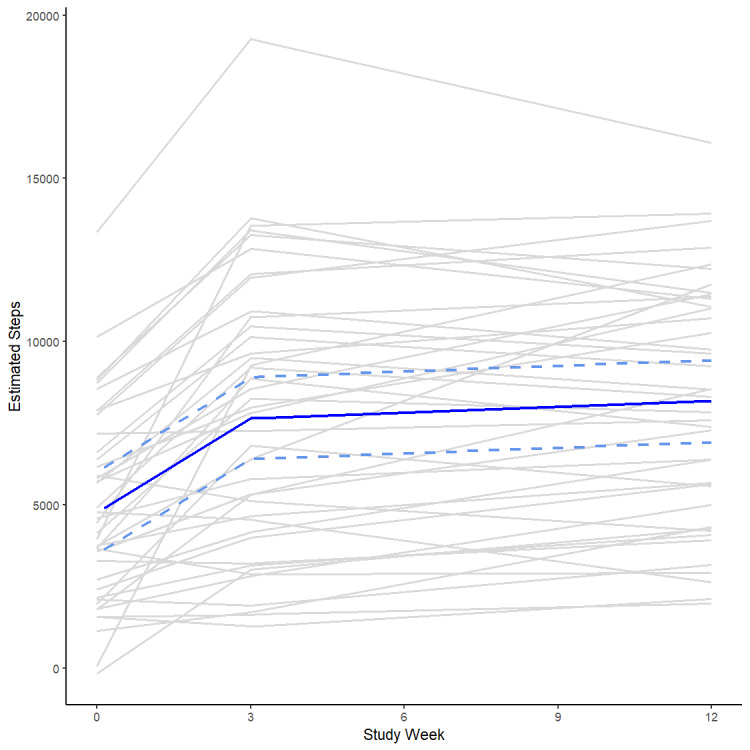
Step count estimates during the 12-week study period from a linear mixed model. Individual participant projections are shown in light grey, and the population mean, with 95% CIs, is shown in blue. To compensate for different fitness levels, a linear mixed model with a random intercept and random slopes before and after the inflection point was used.

Although there was substantial variation in baseline step counts between and within individuals (Multimedia Appendix 1), in general, participants showed a marked improvement in daily step counts during the first 3 weeks, followed by more gradual improvement in the remaining 9 weeks. Initially, step counts increased by an average of 930 (95% CI 547-1312; *P*<.001) steps per week, culminating in an average daily step count of 7658 (95% CI 6257-9059; *P*<.001) at the end of week 3. During the remaining 9 weeks of the study, weekly step counts increased by an average of 67 (95% CI −30 to 163; *P*<.17) steps per week, resulting in a final estimate of 8258 (95% CI 6933-9584; *P*<.001) daily steps (Figure 1). These results were minimally impacted when low step count days (defined as 2 SDs below the participants’ geometric mean) were removed from the analysis.

Covariates that were considered potentially predictive of an increase in daily steps over the 12-week study period were put into a fully adjusted model, including sex, BMI, age, and treatment group. Given substantial variation in steps, differences between treatment groups and sex failed to reach statistical significance. Participants in the control group had a higher average daily step count (mean 866; 95% CI −932 to 2664; *P*=.35) compared to that of the intervention arm. Female participants had 1153 (95% CI −2887 to 581; *P*=.19) fewer daily steps compared to those of male participants. On average, older participants and those with a higher BMI had a lower daily step count. For every 1-year increase in age, the daily step count was lower by 67 (95% CI −159 to 25; *P*=.15) steps on average. Similarly, for every 1-point increase in BMI, the average daily step count was lower by 182 (95% CI −280 to −84; *P*<.001) steps.

**Table 1:** Demographic information (by treatment arm) of participants with available Fitbit data.

		Control group (n=14)	Intervention group (n=27)	All participants (N=41)	*P* value	
Age (years), median (IQR)		52 (45-60)	53 (44-60)	53 (44-61)	.71	
**Sex, n (%)**						.75
	Female	6 (43)	13 (48)	19 (46)		
	Male	8 (57)	14 (52)	22 (54)		
BMI (kg/m^2^), median (IQR)		33 (27-40)	33 (28-38)	33 (28-38)	.91	
**Race, n (%)**						.36
	Black or African American	1 (7)	4 (15)	5 (12)		
	White	7 (50)	17 (63)	24 (59)		
	Other or multiracial	6 (43)	6 (22)	12 (29)		
**Ethnicity, n (%)**						.17
	Hispanic or Latino	6 (43)	6 (22)	12 (29)		
	Not Hispanic or Latino	8 (57)	21 (7)	29 (71)		
Hospital stay (days), median (IQR)		6 (3-8)	4 (2-7)	4 (2-8)	.16	
**Admitted into the hospital intensive care unit, n (%)**						.39
	Yes	2 (14)	7 (26)	9 (22)		
	No	12 (86)	20 (74)	32 (78)		
**Steps in week 1**						.09
	Missing data, n	1	2	3		
	Number of steps, median (IQR)	6492 (4000-7783)	4412 (2274-6536)	4928 (3083-7574)		

## Discussion

### Principal Findings

This study demonstrates that the largest improvements in step counts following recovery from COVID-19 occurred during the first 3 weeks after study enrollment. Both the intervention group and the control group demonstrated similar improvements in step count recovery. Despite the fact that the control group did not engage in any formal structured rehabilitation, the materials (including handouts, devices, and an app with a health diary) that were provided to the control group and the regular check-ins with the study team may help to explain the similar between-group results [7]. In the Hunter et al [1] study on the recovery of COVID-19 survivors, participants indicated that smartwatches motivated them to recover and increase their physical activity levels. This is important, as the use of smartwatches is a relatively low-cost, low-burden, and easily implementable posthospital intervention.

Our study is among the first to report objective physical activity data (step counts) from COVID-19 survivors. In the only other study that we are aware of, Hunter et al [1] found that individuals who were recovering from COVID-19 and were admitted to a critical care unit had an average of 4359 (SD 3488) steps per day in the first month after discharge and increased their step counts to an average of 7914 (SD 4146) steps per day at 1 year (*P*=.003) [1]. In our study, participants had a similar median baseline step count of 4928 (IQR 3083-7574) at 1 week after study enrollment (mean 3.5 weeks after hospital discharge), which is below the targets for healthy adults (around 8000-10,000 daily steps) [8]. Our participants’ average daily step count was 7658 at 3 weeks after study enrollment, which is very similar to the 1-year follow-up estimates (mean 7914, SD 4146 steps) reported by Hunter et al [1], suggesting that the baseline level of activity may be achieved relatively quickly and maintained over the subsequent year. Like our study, Hunter et al [1] included an interventional component in which a subgroup of the cohort had their smartwatch data reviewed monthly by a multidisciplinary team, and rehabilitation goals were remotely communicated to the patients. This may have influenced step count recovery estimates for the overall cohort. An important distinction to note is that our study population was not restricted to patients who were admitted to the hospital and required critical care and ventilation [1]. Therefore, it is possible that the step count recovery in our study was accelerated in comparison and was more representative of a general population of individuals recovering after hospital discharge.

A limitation of our data set is that we do not have metrics regarding wear time for those who had Fitbit devices; thus, our daily average step counts may not reflect true activity and may have been underestimated. Moreover, this trial occurred at a single center in Colorado; therefore, our results are most generalizable to similar populations. Finally, our sample is small, and a 12-week follow-up is considered short-term. However, given that this is the first study to report post–hospital discharge step count data from a heterogeneous group of COVID-19 survivors, we believe that our results are noteworthy.

### Conclusion

Our findings, in combination with prior studies, suggest that physical activity data and step count recovery trajectories may be considered surrogates for physiological recovery, although further research is needed. Further, while activity trajectories may differ based on the presence or absence of post–COVID-19 conditions, monitoring changes over time can provide immediate, objective feedback to patients and support patient-specific goals in the return to prior levels of function following a hospitalization.

## Appendix

Multimedia Appendix 1 Daily Fitbit step counts for individuals over the duration of the study. Low step count days, in which the Fitbit device may not have been worn consistently, are highlighted.

## Abbreviations

AFTER: App-Facilitated Tele-Rehabilitation
