# Supplementary material for: Using Wearable Technology to Quantify Physical Activity Recovery: Secondary Report From the AFTER (App-Facilitated Tele-Rehabilitation) Program for COVID-19 Survivors Randomized Study
Source: JMIR Rehabil Assist Technol. 2023 Mar 20;10:e43436. doi: 10.2196/43436 (PMC10131697; doi:10.2196/43436)
Supplement: Supplementary file 1 [file rehab_v10i1e43436_app1.docx]

**Supplemental Figure 1**. Daily Fitbit step counts for individuals over the duration of the study. The range of the y-axis varies by individual. Low step counts days, in which the Fitbit device may not have been worn consistently, are highlighted.*

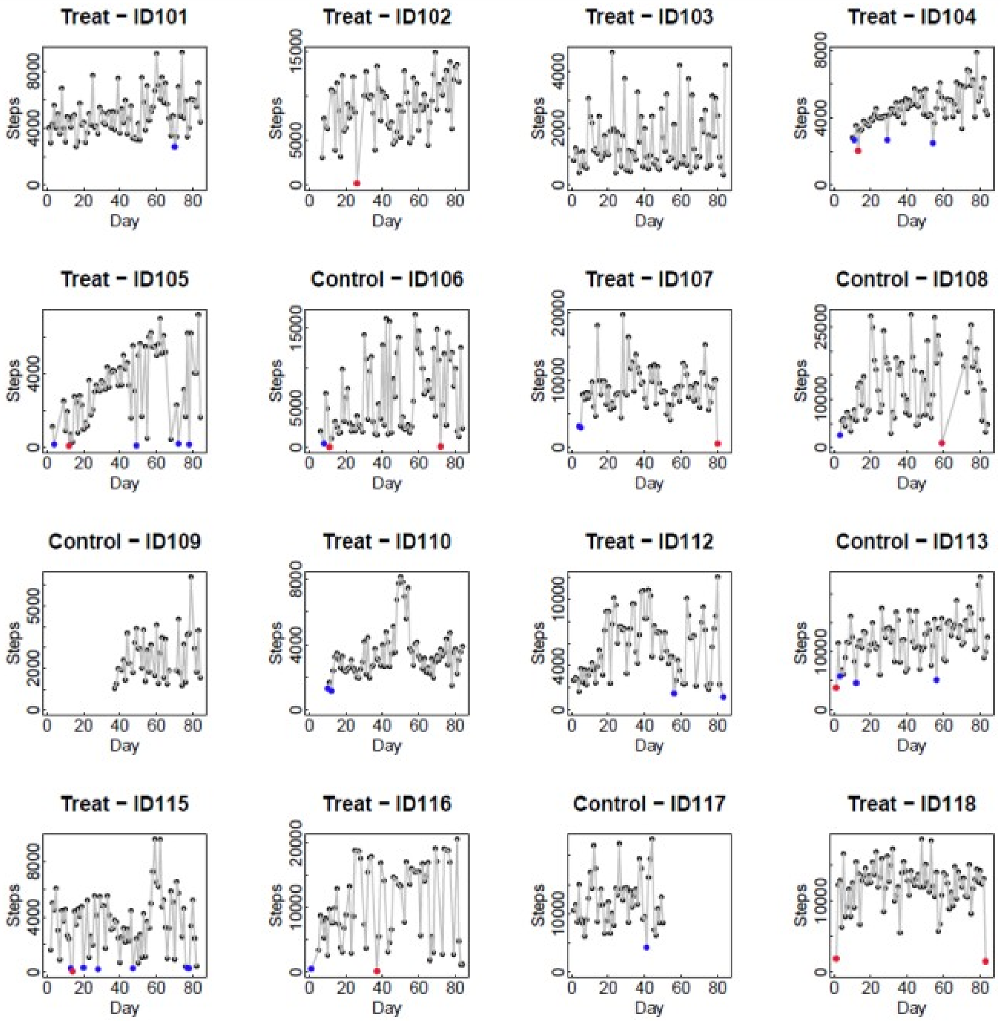

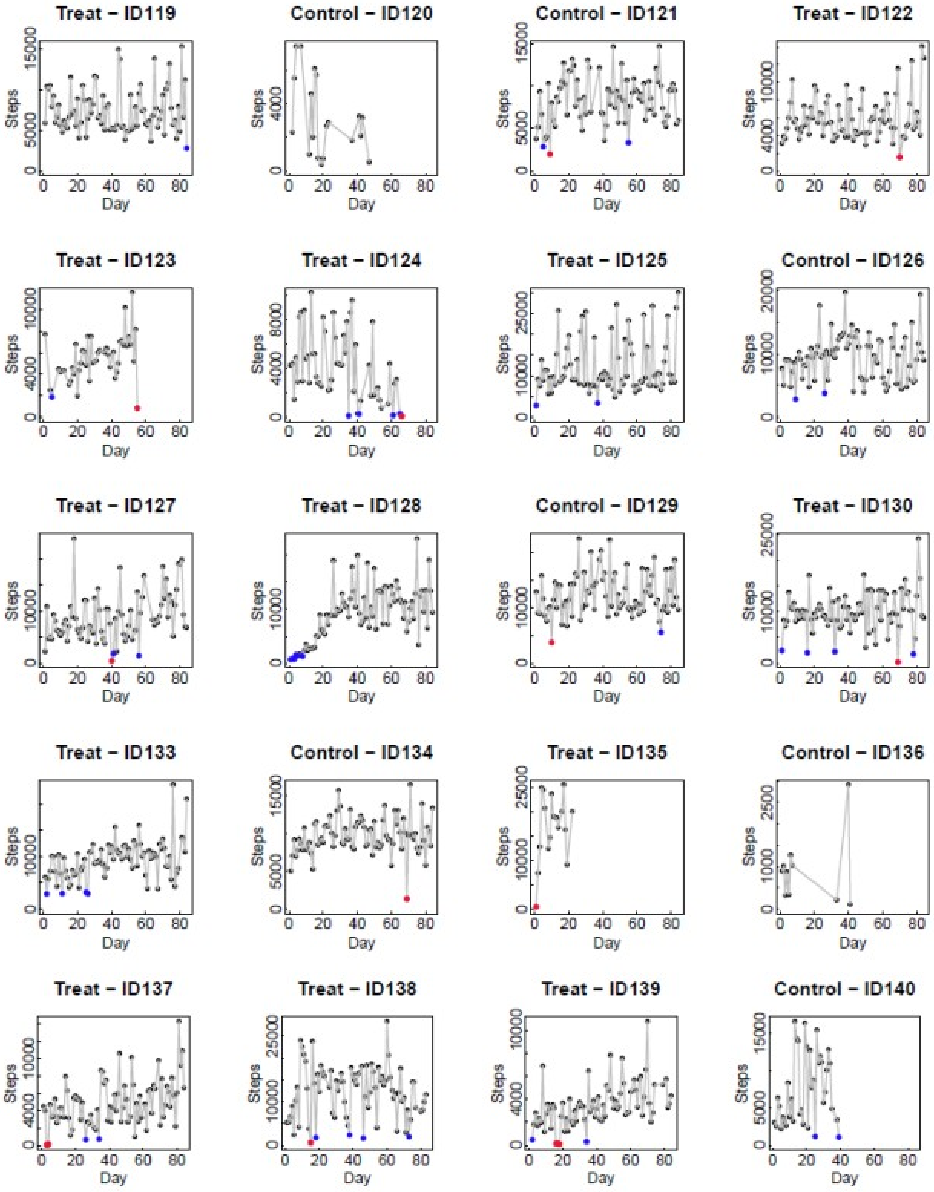
Treat – ID119
Control – ID120
Control – ID121
Treat – ID122
Treat – ID123
Treat – ID124
Treat – ID125
Control – ID126
Treat – ID127
Treat – ID128
Control – ID129
Treat – ID130
Treat – ID133
Control – ID134
Treat – ID135
Control – ID136
Treat – ID137
Treat – ID138
Treat – ID139
Control – ID140
Steps
Day

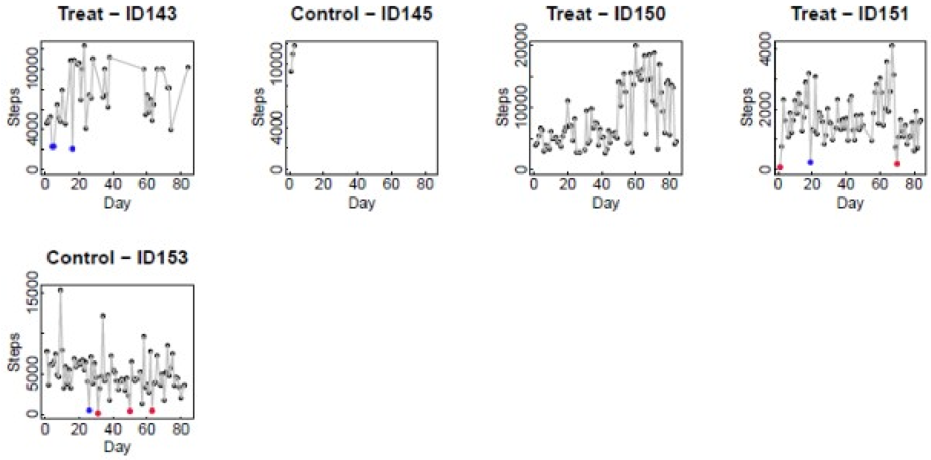


* Colored points represent 2 (blue) or 3 (red) standard deviations away from the individual's geometric mean.
